# Nonlinear swing dynamics of tower crane load subject to stochastic wind excitation: A CFD/CSD coupling approach

**DOI:** 10.1371/journal.pone.0342197

**Published:** 2026-04-02

**Authors:** Yu Sun, Xinhui Zhang, Peijin Liu, Qi Bian

**Affiliations:** 1 School of Science, Xi’an University of Architecture and Technology, Xi’an, Shaanxi, China; 2 School of Mechanical and Electrical Engineering, Xi’an University of Architecture and Technology, Xi’an, Shaanxi, China; Tongji University, CHINA

## Abstract

With the expanding deployment of tower cranes in high-altitude environments, the dynamic interference of stochastic wind excitation on load swing has become a critical concern. Conventional crane dynamic models, limited by assumptions of constant wind excitation and fully rigid crane structures, inadequately capture the multi-physics coupling effects among stochastic wind, flexible tower jib, and load swing, resulting in unreliable swing predictions under wind disturbances. To overcome these limitations, this study innovatively establishes a two-way fluid-structure coupling framework integrating tower crane multi-body dynamic model and full-direction wind field model to simulate wind-induced load swing during crane operations. Based on this framework, this study quantitatively reveals load swing behavior during luffing/slewing operations under stochastic wind excitation by clarifying the wind-structure-load coupling mechanism, specifically incorporating effects of time-varying windward pressure and tower jib wind-induced vibration. The results demonstrate that the time-varying windward pressure distribution(the error exceeds 10%) and tower jib wind-induced vibration significantly influence load swing(the maximum offset is 1.82°). Wind speed/direction variations induce obvious behavior deviations in load swing during crane operations, and there is a significant correlation between the changing trend in radial/tangential swing angles of the load under different wind directions. Using two-way fluid-structure coupling, this study quantifies the nonlinear swing behavior of tower crane load subjected to stochastic winds. The revealed mechanisms provide a quantitative basis for developing environment-adaptive anti-swing controllers and high-precision positioning systems in intelligent tower cranes.

## 1. Introduction

The advancement of China’s “Intelligent Construction” strategy has positioned autonomous tower cranes as pivotal equipment driving the industry’s intelligent transformation [1,2]. Their operational stability and safety critically influence construction efficiency and on-site risk mitigation safety. However, as tower cranes grow in size and operate more frequently at high altitudes with increasing structural dimensions and high-altitude operations [3], the dynamic coupling between stochastic wind excitation and load swing poses significant challenges [4]. Autonomous tower cranes must simultaneously address multi-directional wind excitation and suppress load swing, which requires elucidating the dynamic coupling mechanism among wind excitation, flexible tower jib, and load swing under stochastic wind excitation. Resolving this issue is not only crucial for developing environment-adaptive swing control algorithms but also an urgent requirement for overcoming the safety limitations of autonomous intelligent tower cranes [5,6].

Current research on the inherent swing of tower cranes during acceleration/deceleration phases has established a relatively comprehensive theoretical framework [7]. Models based on Lagrange equations have demonstrated strong applicability in predicting load swing during these phases. Li et al. [8] constructed a seven-degree-of-freedom slewing crane system model. successfully analyzing the coupling between jib motion and spherical swing behavior of the load. Liu et al. [9] established a rigid-flexible-electromechanical coupling model of tower cranes, which can accurately simulate load swing caused by wire rope flexibility and nonlinear electromechanical interactions. However, traditional modeling methods generally have two limitations: (1) Treating the crane structure as a rigid body, ignoring the secondary excitation effect of flexible tower jib vibration response on load swing caused by wind excitation; (2) Assuming that the tower crane is operating in an ideal environment, thereby ignoring the influence of stochastic wind excitation on load. Despite the innovative multi-body dynamics model proposed by Li et al. [10], which effectively reduces load swing by accounting for structural vibrations and utilizing cable-length adjustment, their work overlooks a critical environmental factor: the impact of stochastic wind excitation. The neglect of these two dynamic elements causes a systematic deviation between theoretical prediction and actual working conditions, which directly restricts the improvement of the anti-swing control accuracy of the intelligent tower crane and the applicability of the engineering.

In the study of the mechanism of tower crane affected by wind excitation, the existing results show significant field limitations due to differences in subject focus. The field of structural engineering mainly focuses on the analysis of wind vibration response of the tower crane body, Liu et al. [11] establish a three-dimensional numerical wind tunnel model under the all-wind angle condition, decompose the stochastic wind field into transverse and lateral wind load components, and the system reveals the non-stationary behavior of the aerodynamic load of the special-shaped cross-sectional structure of the tower crane. Chen et al. [12] studied the wind vibration response of four typical wind directions, and revealed for the first time the behavior of tower cranes producing maximum displacement, bending stress and axial stress under the action of forward wind, and proposed a dynamic wind excitation safety assessment plan based on CFD and autoregression method, providing a key basis for the wind resistance design of tower cranes under multi-wind coupling. However, these studies mainly focus on the safety assessment of tower crane structure, and lacks the secondary excitation effect of the structural wind vibration response transmitted to the load through the lifting system. While advanced methods from related fields (e.g., analysis of translational-torsional coupling in asymmetric buildings under seismic loads [13] and probabilistic assessment of wind effects on slender bridges [14]) offer valuable insights for analyzing stochastic loads, they have not been systematically applied to model the coupled “wind-structure-payload” dynamics specific to tower cranes.

The field of crane dynamics focuses on the direct impact of wind excitation on load swing, Abdullahi et al. [15,16] studied the load swing of crane lifting operations under wind-load disturbance, established a dynamic model that considers environmental wind disturbance, and initially revealed the correlation behavior between instantaneous load swing and residual swing. C. Hou et al. [17] established a nonlinear dynamic model of the 5-degree of freedom tower crane, and studied the influence of factors such as load mass, friction and wind disturbance on the load swing of the tower crane.

However, these studies simplify the wind excitation as static pressure acting directly on the load surface [18], the nonlinear time-varying characteristics of wind pressure caused by changes in load space and posture during tower crane operation are not considered. The splitting between fields makes it difficult for existing research to systematically build a dynamic coupling mechanism of “wind -structure-load”: structural engineering overlooks the dynamic response of load, while crane dynamics research ignores the coupling between flexible crane structures and wind fields. This limitation directly restricts the accurate prediction of the three-dimensional swing trajectory of the load of the random air-loaded tower crane.

The stochastic nature of wind excitation lies in its spatiotemporal uncertainty—time-varying wind speeds and stochastic spatial distribution of wind directions [19,20]. JIN L et al. [21] established a soft cable swing model for tower crane swing model. Through this model, the movement of slings and the fluctuation rules of slings at different moments were studied, and the ultimate wind speed was given. Dong et al. [22] developed a dynamic model for gantry cranes incorporating wind excitation, showing that load swing is driven by both trolley motion and wind excitation. While wind excitation shifts the swing’s equilibrium position, they do not alter its period; headwind amplifies swing amplitude, whereas tailwind suppresses it. However, the above research is mainly on the influence of lateral wind excitation on load swing, focusing on the analysis of the wind-induced swing of the load in the two-dimensional plane under a single luffing operation, while the influence of lateral wind excitation and the nonlinear change of the three-dimensional load swing trajectory under slewing operation have not been fully studied. This significant spatial dimension simplification makes it difficult to support the precise swing suppression control requirements of tower crane operations under complex wind field.

Current research on stochastic wind-induced load swing fails to capture the wind-structure-load dynamic coupling mechanism, limiting accurate prediction of nonlinear swing behavior across full-direction wind filed. Therefore, this study takes the QTZ55 tower crane as the research object and proposes a two-way CFD/CSD (Computational Fluid Dynamics/ Computational Structural Dynamics) coupling framework. The specific contributions are as follows:

(1) Overcoming traditional assumptions of constant wind excitation and structural rigidity by integrating the flexible characteristics of wire rope, jib wind vibration and time-varying windward pressure.
(2) Systematically exploring the dynamic coupling mechanism of wind-jib-load in the multi-degree of freedom coupling system of tower cranes,
(3) Comprehensive parametric analysis revealing the influence of wind speed, wind direction, and crane operation mode on load swing characteristics.

The above contributions provide scientific insights for adaptive swing control and dynamic safety thresholds in intelligent cranes.

## 2. Multiphysics coupled modeling method

This section elaborates on how this study overcomes the limitations of conventional tower crane modeling methods (such as the difficulty in solving high-order differential equations and the inability to adequately capture multi-physics coupling effects among stochastic wind, flexible tower jib, and load swing), and proposing a two-way fluid-structure coupling framework that integrates a multibody dynamic model of the tower crane with a full-direction wind field model to simulate wind-induced load sway during crane operation, with key steps including the multibody dynamic model, the full-direction wind field model, and the two-way coupling connection, thereby ensuring the transparency and reproducibility of the subsequent research results.

### 2.1. System description and key assumptions

This study takes QTZ55 tower crane as the research object. As the mainstream equipment in the domestic construction field, it has both structural dynamic characteristics and stochastic wind field interaction mechanism, which are typical and universal [23].

**Fig 1:**
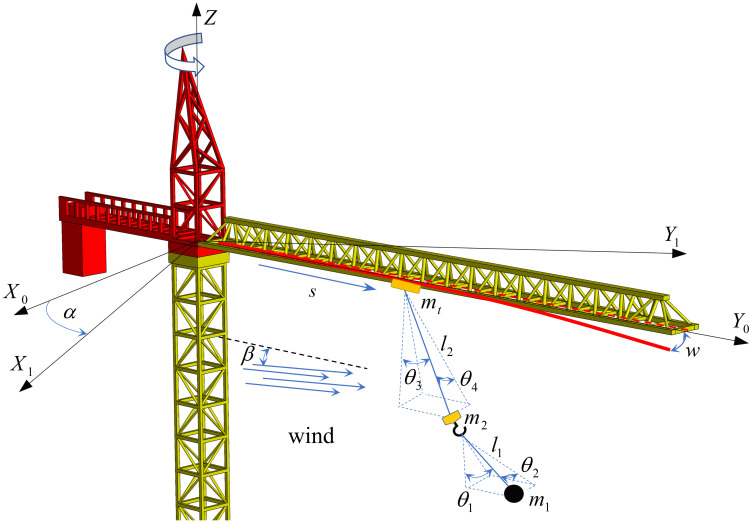
Dynamic model of the tower crane under wind excitation.

As shown in Fig 1, the definitions and assumptions are as follows:

Define the coordinate system and direction of movement: X-axis (radial swing), Y-axis (tangential swing), Z-axis (counterclockwise slewing).

Working operation: luffing operation (the trolley moves along the jib away from the tower body), slewing operation (the tower jib rotates around the tower body).

Flexible characteristics: tower jib deflection *w*, wire rope (hook-to-load *l*_1_, hook-to-trolley *l*_2_).

Load swing behavior: load mass *m*_1_, hook mass *m*_2_, load double swing angle (the radial swing angle of the load and the hook $\theta_{\text{1}}$, $\theta_{\text{3}}$ and tangential declination $\theta_{\text{2}}$, $\theta_{\text{4}}$).

Wind excitation parameters: wind speed *v*_*w*_, wind direction angle β*.*

Model simplification assumptions: Ignore the elastic deformation of secondary structures such as towers and hooks (The jib is modeled as a flexible body to capture its wind-induced vibration); ignore the nonlinear friction, backlash and other dynamic characteristics of motors and gearboxes; assume that the thermal expansion and contraction of metals have no significant effect on stiffness and rope length.

The key structural parameters and material properties are shown in Table 1 and Table 2 (The parameters of Table 2 are partly derived from national standards and industry specifications such as the materials used in parts and components, Poisson’s ratio, and partly from the model and material of the equipment used such as the tensile strength of the wire rope. The remaining parameters are the default parameters in the simulation software Ansys).

**Table 1:** Tower crane structure parameters.

Parameter	Value	Unit	Parameter	Value	Unit
Tower height	48.9	m	m_1_	1000	kg
Jib length	55	m	m_2_	50	kg
Counter-jib length	12.4	m	m_t_	500	kg
Basic unit size	4.5 × 4.5 × 1.4	m	*l*_1_	2.5	m
Initial slewing radius	25	m	*l*_2_	10	m
Wire rope diameter	13	mm	Jib mass	32000	kg

**Table 2:** Tower crane material parameters.

Structure	Material	Density(kg/m³)	Tensile yield strength (MPa)	Poisson’s ratio	Young’s modulus (MPa)
Jib	Structural steel	7850	250	0.29	2 × 10^5^
Wire rope	Carbon steel	7850	1500	0.3	2.5 × 10^5^
Load	Concrete	2300	5	0.18	3 × 10^4^

### 2.2. Wind-tower jib-load energy transfer path

**Fig 2:**
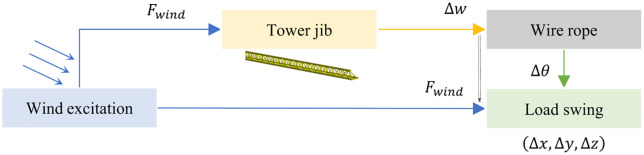
Wind-Tower jib-load energy transfer.

Based on the fragmentation between structural wind-response and wind-load response studies highlighted in the introduction, this section elucidates their intrinsic correlation through the physical mechanism of wind energy transmission paths (e.g., Fig 2), kinetic energy conversion from tower jib vibration to load swing), establishing a theoretical bridge for coupled dynamics analysis.

The influence of wind excitation on the load swing is mainly the force of wind excitation acting on the windward surface of the tower crane. The wind-carried energy input is derived from the kinetic energy of the wind-carried, and its power expression is:


$$\begin{array}{c} P_{wind}=F_{wind}v=\frac{\text{1}}{\text{2}}\rho C_{p}Av^{\text{3}} \end{array}$$ (1)

Where *F*_*wind*_ is the wind excitation force, *ρ* is the air density (kg/m3), C_*p*_ is the wind energy capture efficiency, A is the windward area of the tower jib (m^3^), and *v* is the wind speed (m/s),

When the wind excitation acts on the tower jib, the wind energy is partially converted into the vibration energy of the tower jib, and the wind-induced vibration equation is:


$$\begin{array}{c} F_{wind}(x,t)=\frac{\partial^{\text{2}}}{\partial x^{\text{2}}}\left(EI\frac{\partial^{\text{2}}\Delta w}{\partial x^{\text{2}}}\right)+m\frac{\partial^{\text{2}}\Delta w}{\partial t^{\text{2}}}+c\frac{\partial \Delta w}{\partial x} \end{array}$$ (2)

Where *w* represents the deflection of the tower jib and *c* represents the damping coefficient of the structure (N ∙ *S/m*).

The tower jib is simplified into a cantilever beam structure, and its vibration energy includes kinetic energy and elastic potential energy:


$$\begin{array}{c} E_{tower}=E_{k,tower}+E_{p,tower}=\int_{\text{0}}^{l}m(x){\left(\frac{\partial (w+\Delta w)}{\partial t}\right)}^{\text{2}}dx+\frac{\text{1}}{\text{2}}\int_{\text{0}}^{l}EI(x){\left(\frac{\partial^{\text{2}}(w+\Delta w)}{\partial x^{\text{2}}}\right)}^{\text{2}}dx \end{array}$$ (3)

Where *m(x)* is the length mass of the jib unit (kg/m), *EI(x)* is the cross-section bending stiffness (N ∙ m^2^), and Δ*w* represents the deflection of the tower jib.

The vibration of the jib is changed by the vibration of the wire rope excited by the trolley, and the kinetic equation is:


$$\begin{array}{c} \frac{d^{\text{2}}(\theta +\Delta \theta )}{dt^{\text{2}}}+\frac{\mu }{m}\frac{d(\theta +\Delta \theta )}{dt}+\frac{g}{l}sin(\theta +\Delta \theta )=-\frac{\text{1}}{l}\frac{d^{\text{2}}(w+\Delta w)}{dt^{\text{2}}}cos(\theta +\Delta \theta ) \end{array}$$ (4)

Where *θ* represents the swing angle of the wire rope (rad), *l* represents the length of the rope(m), and *μ* represents the air damping coefficient of the rope (N ∙ *S/m*). The kinetic energy of the rope is expressed as $E_{k,rope}=\frac{\text{1}}{\text{2}}m{\left(l\dot{\left(\theta +\Delta \theta \right)}\right)}^{\text{2}}$, and the gravity potential energy of the rope is $E_{p,rope}=mgl\left(\text{1}-cos(\theta +\Delta \theta )\right)$.

The swing of the load is directly determined by the swing angle of the rope. Treat the load as a particle, decompose the swing angle into two radial and tangential directions *θ*_1_ and *θ*_2_, and the parametric equation of the swing trajectory is:


$$\begin{array}{c} \left\{ \begin{array}{c} @cx(t)+\Delta x(t)=\text{s}\cdot \text{cos}\alpha -lsin\alpha \cdot \text{sin}{\left(\theta +\Delta \theta \right)}_{\text{2}}+lcos\alpha \cdot \text{sin}{\left(\theta +\Delta \theta \right)}_{\text{1}}\cdot \text{cos}{\left(\theta +\Delta \theta \right)}_{\text{2}}\\ y(t)+\Delta y(t)=\text{s}\cdot \text{cos}\alpha +lcos\alpha \cdot \text{sin}{\theta (\theta +\Delta \theta )}_{\text{2}}+lsin\alpha \cdot \text{sin}{\left(\theta +\Delta \theta \right)}_{\text{1}}\cdot \text{cos}{\left(\theta +\Delta \theta \right)}_{\text{2}}\\ \;z(t)+\Delta \;z(t)=-l\text{cos}{\left(\theta +\Delta \theta \right)}_{\text{1}}\cdot \text{sin}{\left(\theta +\Delta \theta \right)}_{\text{2}} \end{array}\right.\; \end{array}$$ (5)

Where Δ*x*(t), Δ*y*(t), Δ*z*(t) represent the offset of the load swing trajectory over time due to the wind load transmitted to the load through the tower jib and wire rope.

The load swing energy equation is:


$$\begin{array}{c} E_{k,load}=\frac{\text{1}}{\text{2}}m\left({\left(\dot{x}+\dot{\Delta x}\right)}^{\text{2}}+{\left(\dot{y}+\dot{\Delta y}\right)}^{\text{2}}+{\left(\dot{z}+\dot{\Delta z}\right)}^{\text{2}}\right) \end{array}$$ (6)

$$\begin{array}{c} E_{p,load}=mg(z+\Delta \;z) \end{array}$$ (7)

Based on the comprehensive energy transfer path, the energy conservation equation of the system is:


$$\begin{array}{c} E_{kinetic}=E_{k,tower}+E_{k,rope}+E_{k,load} \end{array}$$ (8)

$$\begin{array}{c} E_{potential}=E_{p,tower}+E_{p,rope}+E_{p,load} \end{array}$$ (9)

$$\begin{array}{c} P_{wind}=\frac{d}{dt}\left(E_{kinetic}+E_{potential}+E_{diss}\right) \end{array}$$ (10)

Where the dissipated energy *E*_*diss*_ includes: friction in the material, structural damping, pneumatic dissipation of ropes and load.

In summary, it can be seen that the transmission process of wind energy from the tower jib to the load is achieved through a series of nonlinear interactions: the wind-borne aerodynamic force excites the vibration at the end of the tower jib (Δ*w*), which in turn affects the swing angle of the wire rope (Δ*θ*), and ultimately leads to the offset of the load space trajectory (Δ*x,* Δ*y,* Δ*z*).However, traditional analytical methods face dual challenges: (1) It is difficult to solve high-order partial differential equations (such as the fourth-order partial conduction in the Euler-Bernoulli beam vibration equation) and nonlinear energy dissipation effects; (2) It is impossible to simultaneously capture the composite wind load effect-indirect excitation (transmitted through the tower jib-rope) and direct excitation (time-varying wind pressure on the load surface).In order to break through these limitations, this research adopts a finite element framework, integrates a nonlinear solver, and multi-physics coupling (CFD/fluid mechanics, CSD/structural mechanics, and multibody dynamics) [24].The analysis framework can accurately quantify the nonlinear swing trajectory of the tower crane load under wind load, and realize the accurate prediction of the load trajectory offset in a complex wind load environment.

### 2.3. CFD/CSD coupling analysis framework

**Fig 3:**
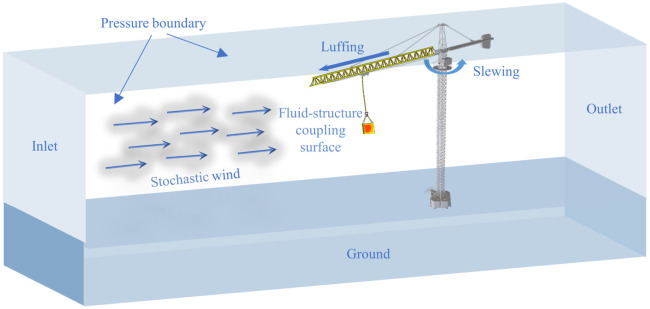
CFD/CSD coupling simulation domain and boundary conditions.

Traditional tower crane dynamic models ignore the dynamic response of flexible structures and the space-time randomness of wind excitation, making it difficult to accurately characterize the nonlinear swing behavior of load. Therefore, this study establishes a two-way fluid-structure coupling framework integrating tower crane multi-body dynamic model and full-direction wind field model to simulate wind-induced load swing during crane operations, the three-dimensional diagram is shown in Fig 3. This model dynamically quantifies the wind-induced vibration of tower cranes during slewing and luffing motions under stochastic wind load. Crucially, the structural response propagates to the suspended load via flexible wire rope deformation, while real-time changes in wind pressure distribution —driven by crane motion (e.g., slewing angle variation)— trigger nonlinear load swing through wind-structure-load coupling.

**Fig 4:**
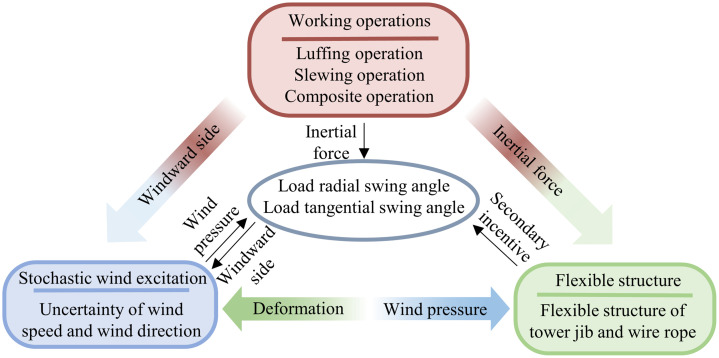
Multiphysics interaction influence mechanism.

The tower crane load swing under stochastic wind excitation exhibits the influence of Multiphysics interaction, as shown in Fig 4. the surrounding flow field is reconstructed and the area of wind pressure is changed. At the same time, the inertial force in the acceleration and deceleration stage of the operation will deform the flexible structure; under different operations, the wind excitation is transmitted through the wind-structure-load coupling, forming a three-dimensional spatial swing trajectory of the load with different characteristics.

**Fig 5:**
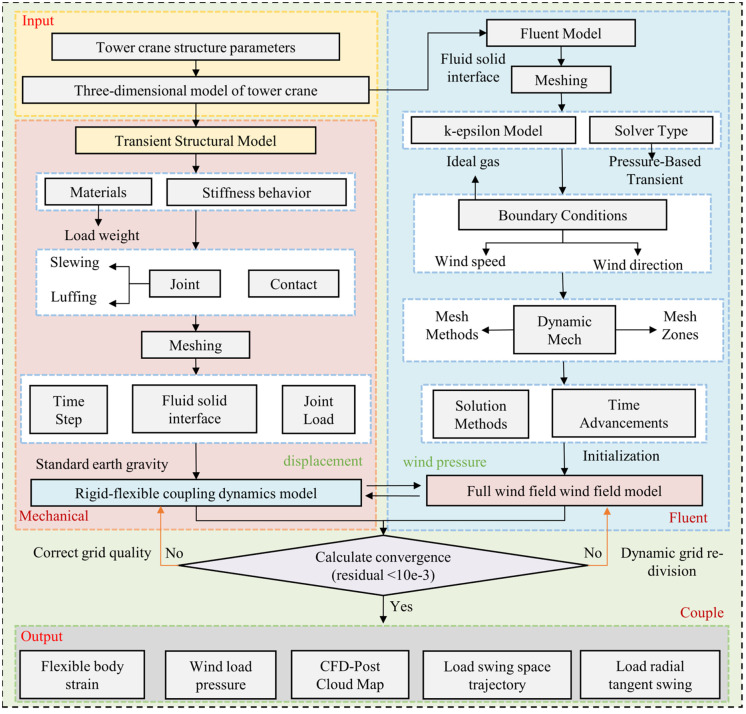
Tower crane CFD/CSD two-way coupling analysis process.

The CFD/CSD two-way coupling analysis process is shown in Fig 5, the specific steps are as follows:

(1) Through the real tower crane structure size parameters, establish the three-dimensional model of the tower crane. The mechanical and Fluent plates are imported separately for the setting of the solid area and the fluid area.
(2) Set the solid material parameters and stiffness behavior in Mechanical, set the connection sub-connection and the contact of parts; mesh the flexible body, and set the gravity, time step, and operating speed and acceleration.
(3) Fluid area meshing, set the boundary of the fluid basin to determine the direction of the wind field; set the turbulence model, wind speed size, and dynamic meshing method in Fluent; set the solution method and time step size and initialize it.
(4) Connect the multibody dynamics model in Mechanical and the wind field model in Fluent to the system coupling module for two-way fluid-solid coupling analysis.
(5) Analyze the swing angle and horizontal displacement of the load in both tangential and radial directions through mechanical post-processing, and analyze the wind pressure and flow field through the CFX post-processing module.

### 2.4. Multi-body dynamic modeling

**Fig 6:**
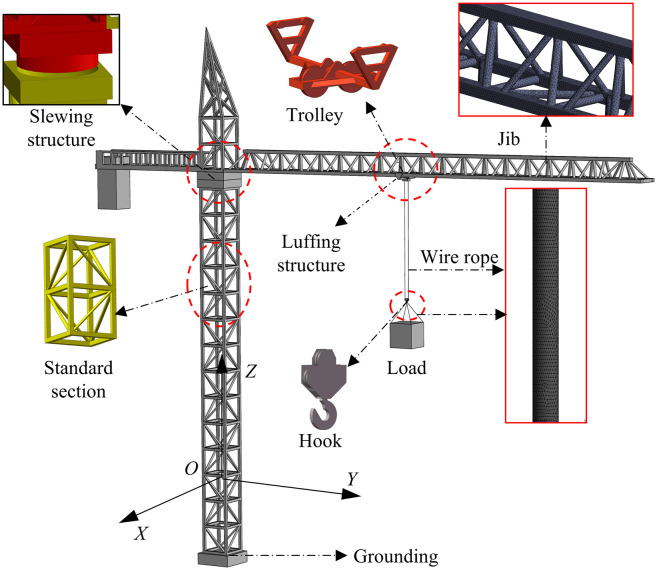
Multibody dynamics model and soft body meshing.

**Fig 7:**
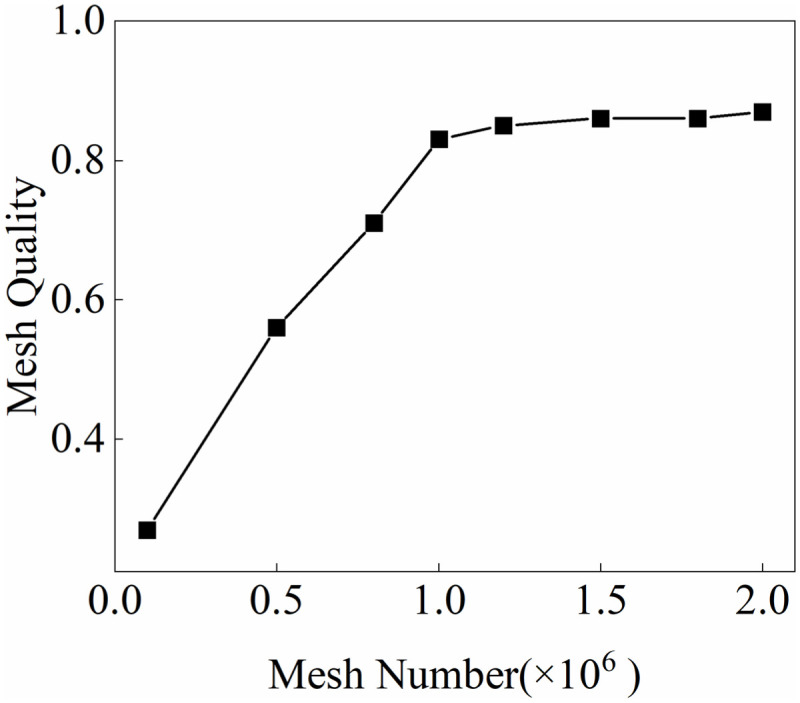
Mesh independence verification results of soft structure area.

This study used ANSYS Mechanical as a structural mechanics (CSD) solver. With wind-load-sensitive structural areas such as tower jib, wire ropes, and load as the core, the solid domain grid is divided by tetrahedron elements that can adapt to complex surface forms. The adaptive size adjustment of the grid encrypts the local surface area by capturing curvature and proximity. As shown in Fig 6, fluid solid interaction area is necessary to perform Boolean operation on the solid area through the wind field area to obtain, and the geometric surface junction is prone to geometric interference problems during the Boolean operation, so the structural part of the tower crane needs to be simplified accordingly to retain key connection areas (e.g., the tower jib slewing connection area, trolley translation). While connecting the area, try to meet the actual tower crane structure characteristics as much as possible. On the premise of ensuring grid quality, by weighing the calculation accuracy and efficiency (unit mass >0.6, aspect ratio <8), to ensure the simulation reliability of structural dynamic response (e.g., the tower jib slewing connection area, trolley translation tower jib and wire rope deformation). The verification results of the mesh independence of the structure area are shown in Fig 7. When the mesh number is 3 million, the quality of the global mesh is about 0.9. Increasing the mesh number has less impact on the mesh quality. Therefore, this study used the 3 million mesh number of the structure area for calculation.

**Fig 8:**
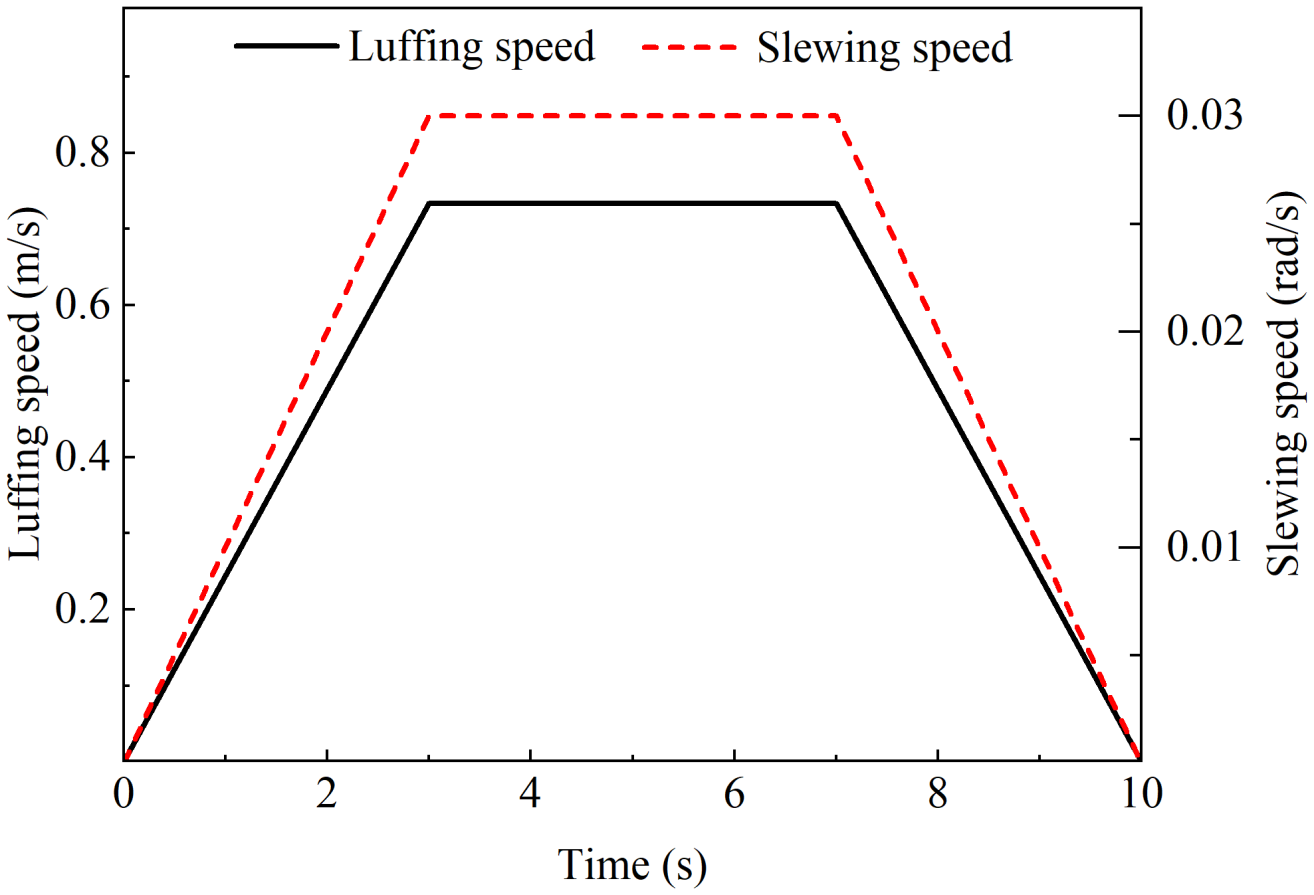
Operation settings.

Under standard earth gravity field, the tower crane solid model adopts frictionless contact constraints, turn off the weak spring option in the solution settings and enables the large deflection and medium speed dynamic settings. The operation process is divided into three stages: luffing system drives the trolley to move along the tower jib at an acceleration of 0.244 m/s² to a constant speed of 0.733 m/s, and then brakes at an equal deceleration. slewing system drives the tower jib at an angular acceleration of 0.01 rad/s² to rotate at a constant speed of 0.03 rad/s, and finally symmetrical deceleration stops. The complete speed-time curve is shown in Fig 8.

### 2.5. Full-direction wind field modeling

**Fig 9:**
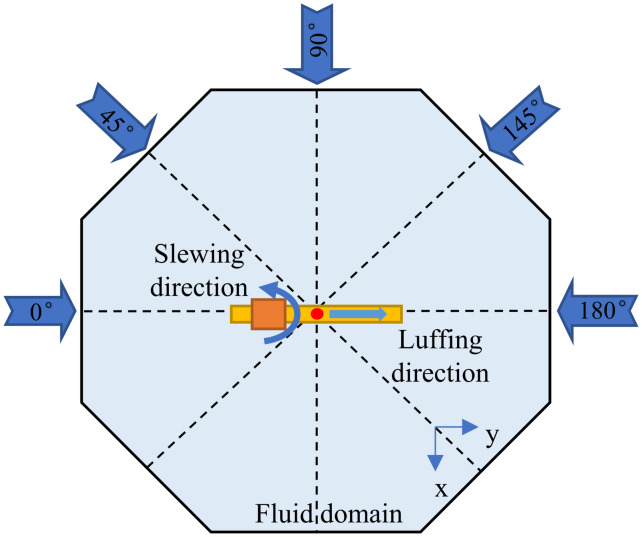
Schematic diagram of the full wind direction angle domain.

**Fig 10:**
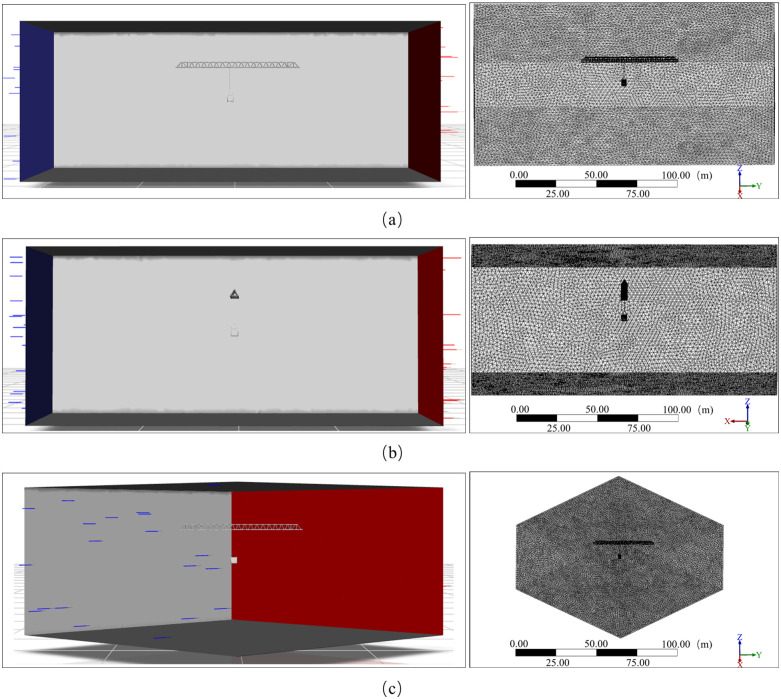
Wind field model and meshing of different wind direction angles. **(a)** 0° and 180° wind angles; **(b)** ±90° wind angle; **(c)** ±45° and ±135° wind angle.

In order to reveal the behavior of tower crane load under the full-direction wind domain, this study used ANSYS Fluent as a computational fluid dynamics (CFD) solver to establish corresponding wind field models (Fig 9) according to different wind angles (Fig 10), through quantification of wind speed time-varying fluctuations and wind direction stochastic distributions, dynamic coupling between wind pressure data and the crane multi-body dynamic model windward surfaces is achieved. The geometric boundary condition is set to: (1) The fluid domain is a cube area of 80m × 70m × 180m. The wind direction is changed by setting the opposite two sides as the wind loading port and the wind loading outlet, the remaining sides are set as walls. (2) The surface of the tower crane is set with the tower jib, wire rope and load as the fluid-solid coupling surface for mutual transmission of fluid mechanics and structural dynamics data. By setting whether the load surface is a fluid-solid coupling surface, to study the influence of the time-varying characteristics of wind pressure on the surface of the lifting weight on the swing of the load.

**Fig 11:**
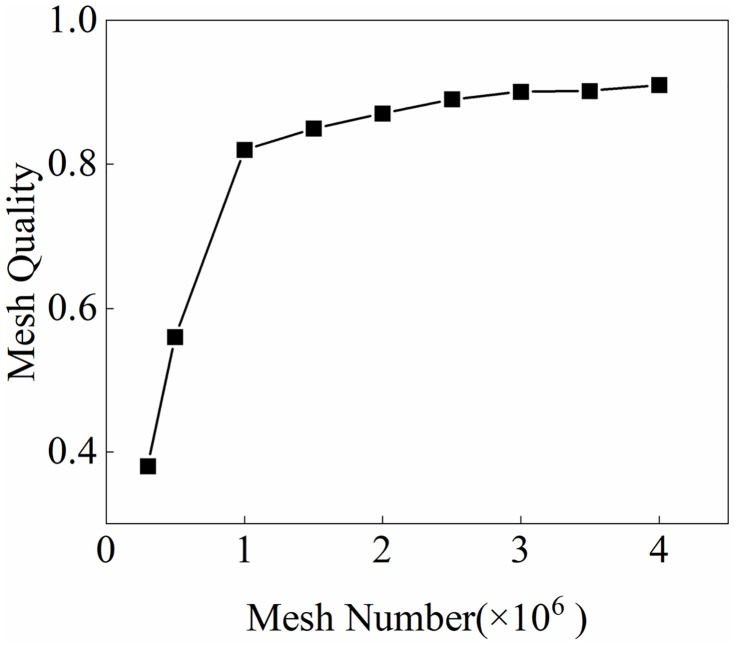
Mesh independence verification results of fluid area.

The wind field meshing of different wind direction angles is shown in the figure on the right of Fig 10. In order to improve the overall grid quality while improving operating efficiency, the wind load sensitive areas such as tower jibs, wire ropes and lifting weights are meshed separately to ensure that the minimum orthogonal mass of the mesh area is > 0.15, the maximum aspect ratio is < 5. The verification results of the mesh independence of the fluid area are shown in Fig 11. When the mesh number is 3 million, the quality of the global mesh is about 0.9. Increasing the mesh number has less impact on the mesh quality. Therefore, this study used the 3 million mesh number of the fluid area for calculation.

A pressure-based transient solver is employed for flow field analysis. To achieve real-time data exchange for two-way fluid-structure coupling, the fluid domain incorporates a dynamic mesh system that responds to displacements of computational boundaries (e.g., tower jib, load). This approach dynamically adjusts mesh topology to synchronize fluid domain updates with moving components, maintaining node-to-node matching at FSI interfaces to eliminate force transfer errors. Concurrently, local cell reconstruction updates the dynamic mesh every 1 s, with the region type set to System Coupling. Mesh deformation utilizes the Diffusion smoothing method to achieve gradient-driven smooth transitions, ensuring consistent mesh quality [25].

In the fluid domain, air flow can be described by the continuous equation, the momentum conservation equation, and the energy conservation equation, which are expressed as follows:


$$\begin{array}{c} \frac{\partial \rho }{\partial t}+\frac{\partial }{\partial x_{i}}\left(\rho v_{i}\right)=\text{0} \end{array}$$ (11)

$$\begin{array}{c} \frac{\partial }{\partial t}\left(\rho v_{i}\right)+\frac{\partial }{\partial x_{j}}\left(\rho v_{i}v_{j}\right)=-\frac{\partial P}{\partial x_{i}}+\frac{\partial }{\partial x_{j}}\tau_{ij} \end{array}$$ (12)

$$\begin{array}{c} \frac{\partial }{\partial t}(\rho E)+\frac{\partial }{\partial x_{i}}\left[v_{i}(\rho E+\rho )\right]=\frac{\partial }{\partial x_{i}}\left(K_{eff}\frac{\partial T}{\partial x_{j}}\right)+\frac{\partial }{\partial x_{j}}\left(v_{i}\tau_{ij}\right) \end{array}$$ (13)

Where, *t* is the time (s); $\tau_{ij}$ is the viscous stress; *E* is the total energy (J); *T* is the air temperature (K).

**Fig 12:**
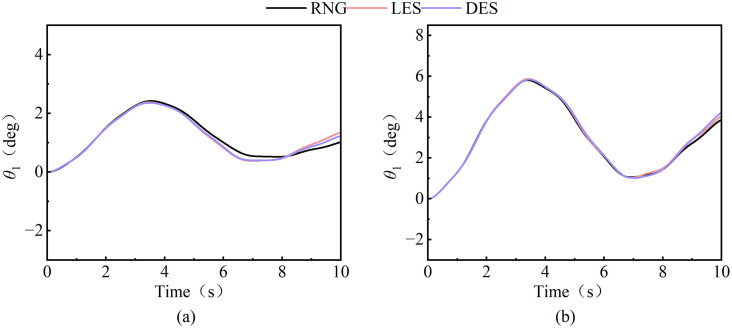
Comparative assessment of RNG, LES, and DES models for predicting wind-induced load swing behavior. **(a)** 6m/s wind excitation; **(b)** 10m/s wind excitation.


In the simulation of tower crane aerodynamics, the selection of a turbulence model requires a careful balance between predictive accuracy and computational cost. While Large Eddy Simulation (LES) and Detached Eddy Simulation (DES) are theoretically superior for resolving unsteady vertical structures under strong gusts, a comparative analysis (see Fig 12) of the predicted load swing angle reveals that the time histories from the RNG, LES, and DES models are in close agreement. The trajectories and amplitudes of the swing show negligible differences (the angle deviation is within 5%), with variations consistently within a narrow band. However, the computational expense associated with the LES and DES models—characterized by significantly longer simulation times and substantially larger storage requirements—is considerably higher. Therefore, given the comparable predictive performance for this specific application and the paramount importance of computational efficiency for practical engineering analysis, the RNG model, which is well-suited for flows with moderate swirl and high strain rates [26], is retained as the optimal and most resource-effective choice for this study. The governing equations of the RNG k-*ε* model are as follows:

$$\begin{array}{c} \frac{\partial (\rho k)}{\partial t}+\frac{\partial \left(\rho kv_{i}\right)}{\partial x_{i}}=\frac{\partial }{\partial x_{j}}\left[\left(\mu +\frac{\mu_{t}}{\sigma_{k}}\right)\frac{\partial k}{\partial x_{j}}\right]+G_{k}-\rho \varepsilon \end{array}$$ (14)

$$\begin{array}{c} \frac{\partial (\rho \varepsilon )}{\partial t}+\frac{\partial \left(\rho \varepsilon v_{i}\right)}{\partial x_{i}}=\frac{\partial }{\partial x_{j}}\left[\left(\mu +\frac{\mu_{t}}{\sigma_{\varepsilon }}\right)\frac{\partial \varepsilon }{\partial x_{j}}\right]+\overset{*}{\underset{\text{1}\varepsilon }{C}}\frac{\varepsilon }{k}G_{k}-C_{\text{2}\varepsilon }\rho \frac{\varepsilon^{\text{2}}}{k} \end{array}$$ (15)

Where *k* is the turbulent kinetic energy, *ε* is the turbulent dissipation rate, μ is the dynamic viscosity, *G*_*k*_ is the turbulent kinetic energy generation term, G_k_ = *μ*_t_*S*^2^, *S* is the strain rate tensor modulus, *μ*_t_ is the turbulent viscosity, *μ*_t =_ *ρC*_*μ*_*k*^2^/ε, C_*μ*_ = 0.0845, $\overset{*}{\underset{\text{1}\varepsilon }{C}}$ is the key improvement term, $\overset{*}{\underset{\text{1}\varepsilon }{C}}=C_{\text{1}\varepsilon }-\left[\begin{array}{c} \eta (\text{1}-\eta /\eta_{\text{0}}) \end{array}\right]/\left(\text{1}+\beta \eta^{\text{3}}\right)$, *η = Sk/*ε, η_0_ = 4.38, β = 0.012, σ_ε_ = 0.7104, *C*_1*ε*_, *C*_2*ε*_, σ_*k*_ is the empirical coefficient of the turbulence model. In this article, the turbulence intensity is set at 5% and the turbulence viscosity ratio is 10.

### 2.6. Load swing characterization metrics

To unravel the spatiotemporal characteristics of the load swing under coupled stochastic winds and crane motions, this study decomposes the load swing into radial and tangential components, evaluates their dynamic behaviors through amplitude, energy, and correlation metrics, and systematically explores wind parameter effects.

#### 2.6.1. Directional decoupling.

**Fig 13:**
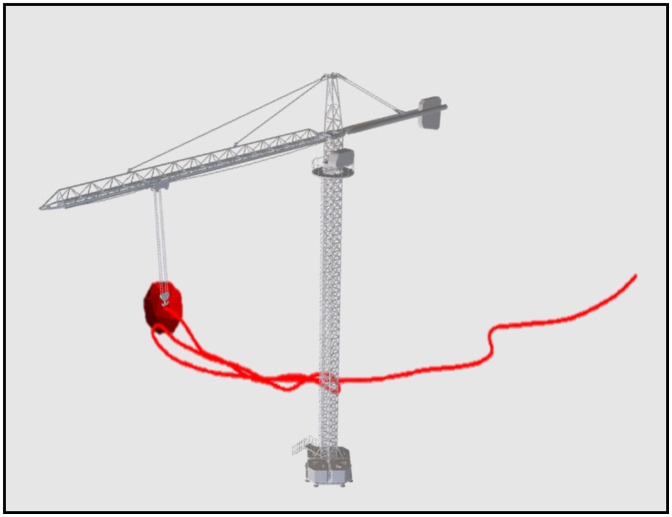
Load space swing trajectory.

The Fig 13 shows the spatial swing trajectory of the load during the operations of the wind-load tower crane. It can be seen that its trajectory is complex and it is difficult to extract regular conclusions. In order to reveal the load swing behavior of the random wind-load tower crane, this paper projects the three-dimensional swing trajectory of the load onto the radial/tangent plane for directional research. This decomposition method can clearly identify the dominant direction of the intensified load swing (e.g., larger *θ*_1_ in luffing motion), thereby providing a more targeted basis for the suppression of the swing for the control system.

#### 2.6.2. Stability metric (RMS).

Unlike conventional peak-focused analyses, the RMS values are calculated to quantify the average swing energy. The RMS of the load swing angle is a key indicator to quantify the dynamic characteristics of the swing. Its kinetic significance reflects the statistical average value of the swing energy, which is directly related to the kinetic energy of the system, and comprehensively reflects the overall intensity of the swing. The more intense the load swing, the greater the RMS value. The RMS expression is as follows:


$$\begin{array}{c} \theta_{RMS}=\sqrt{\frac{\text{1}}{T}\sum_{\text{0}}^{T}\theta^{\text{2}}(t)} \end{array}$$ (16)

With *θ*(t) denoting the instantaneous swing angle and *T* the observation period. The RMS magnitude correlates positively with oscillation intensity, providing a statistical measure of the integrated kinetic energy $E_{k}=\text{1}/\text{2}I{\dot{\theta }}^{\text{2}}$ (where *I* is the moment of inertia), thereby holistically describing the global vibrational energy profile.

## 3. Result and discussion

This section aims to present and provide an in-depth analysis of the mechanism and patterns of the load swing behavior of a tower crane under wind excitation. Its structure is organized as follows: First, the accuracy and reliability of the proposed two-way fluid-structure interaction model are evaluated by comparing the results with existing experimental data from a tower crane model. Subsequently, the mechanism of wind-induced load behavior is analyzed through comparative experiments combined with CFD-Post post-processing results, clarifying the dynamic transfer path of wind-structure-load interactions. Finally, by comparing different tower crane operating conditions, wind speeds, and wind directions, the behavior of wind-induced load swing are derived through analysis of the load’s radial/tangential swing angles, *θ*_RMS_ values, and swing angle correlations.

The numerical simulations in this study were performed on a computational system equipped with an Intel Core i5-14600KF CPU (14 cores, up to 5.3 GHz), an NVIDIA GeForce RTX 4060 GPU (8 GB VRAM), 48 GB of DDR5 RAM (6800 MHz), and a combined storage of 2 TB SSD and 10 TB HDD, running on the Windows 11 operating system (Version 23H2).

### 3.1. Verification

To validate the effectiveness of the proposed model, simulation parameters were configured according to the operational conditions of the QTZ5513 tower crane documented in Reference [27] (Table 3). The engineering accuracy and applicability of the model were evaluated through comparative analysis of load behavior during slewing operation. The motion profile was designed with three distinct phases—acceleration, constant velocity, and deceleration—implemented as follows: Acceleration and deceleration durations were set to 5 s each, with uniform acceleration from 0 to 5 s, constant velocity from 5 to 15 s (exclusive), and uniform deceleration from 15 to 20 s, achieving full stop at t = 20 s. This motion planning comprehensively captures dynamic inertial load during startup/braking transitions of crane slewing mechanisms.

**Table 3:** QTZ55 tower crane parameters.

Parameter	Value	Unit	Parameter	Value	Unit
Load mass	800	kg	Wire rope length	10	m
Trolley mass	324	kg	Slewing radius	40	m
Hook mass	50	kg	Acceleration of gravity	9.8	m ∙ s^-2^
Wire rope Poisson’s ratio	0.3	–	Slewing up-acceleration	0.01	rad ∙ s^-2^
Slewing down-acceleration	0.01	rad ∙ s^-2^	Slewing speed	0.05	rad ∙ s^-2^

**Fig 14:**
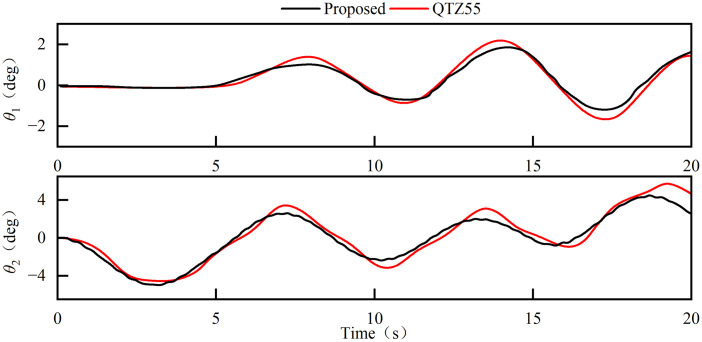
Load swing behavior under luffing operation.

The Fig 14 is a diagram of the comparative analysis results of the results of the two models. It can be seen from the time domain response characteristics that both the radial swing angle and the tangential swing angle curves show significant synchronization, and the phase difference is less than 5%. Quantitative error analysis shows that the simulation deviations of the average radial swing angle and the amplitude are 6.77% and 8.52%, respectively, and the corresponding indicators of the tangential swing angle are 7.43% and 8.65%, respectively. The comparison results show that the deviation between the simulation results and the experimental results is less than 10%. We believe that this is acceptable for characterizing the dynamic behavior of the tower in this engineering study [28]. This verification work provides a reliable theoretical model basis for subsequent research.

### 3.2. Wind-structure-load coupling mechanism

Based on the constructed CFD/CSD two-way coupling analysis framework, this section clarifies the mechanism of wind-structure-load coupling from the two dimensions: stochastic wind and load swing dynamic interaction, stochastic wind and soft jib excitation transmission.

#### 3.2.1. Effects of time-varying windward pressure on load swing.

**Fig 15:**
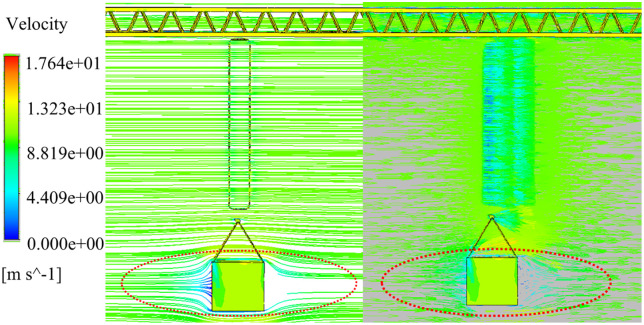
Load windward Contour plot.

The coupling effect of stochastic wind excitation and load swing can be decomposed into the following dynamic processes: (1) The excitation effect of the initial windward surface: the stochastic wind excitation directly acts on the windward surface of the load, causing the initial swing response; (2) The dynamic reconstruction effect of the flow field: the slewing operation of the tower crane and the swing of the load lead to dynamic changes in the load attitude, which in turn changes the wind pressure distribution characteristics of the windward surface. Therefore, this study analyzes the simulation results of fluid-solid coupling through CFD-Post post-processing, and systematically studies the mechanism of action of the dynamic interaction process. The flow field dynamics characteristic analysis cloud diagram in Fig 15 shows that when the wind excitation directly acts on the load-sling system, the windward surface forms a vortex structure with the tangential velocity component shown in the red circle shown in the illustration. The fluid separation effect leads to a nonlinear increase in pneumatic torque.

**Fig 16:**
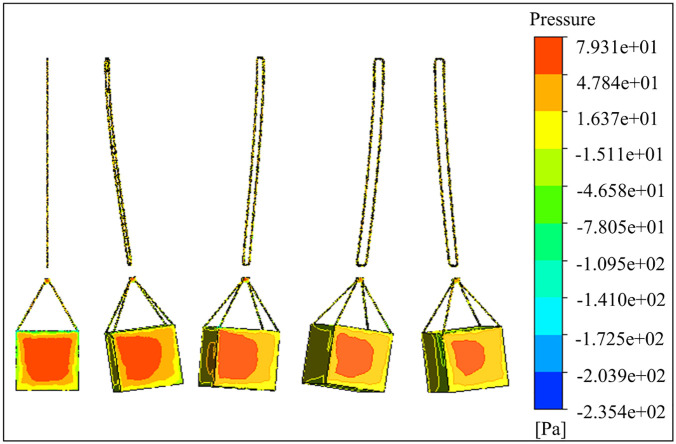
Changes in wind pressure of the load in the slewing operation.

The Fig 16 shows a further analysis of the evolution process of the load transient pressure field: as the tower jib slewing drives the load to swing, the topological structure of the surrounding flow field changes, gradually changing from one-sided load to an asymmetric distribution of bilateral wind load pressure. At the same time, the initial windward surface force area gradually attenuates with the increase of the swing angle. This phenomenon verifies the time-varying characteristics of wind pressure on the windward surface, which provides a key physical basis for the study of the swing law of tower crane load under random wind excitation.

In order to explore the influence of time-varying windward pressure characteristics on load swing, this study quantifies the feedback effect of the time-varying windward pressure characteristics on load swing, compare and analyze the influence of two-way coupling (considering the time-varying characteristics of wind pressure) and one-way coupling (ignoring the time-varying characteristics of wind pressure) on the load swing angle. Table 4 and Table 5 show the behavior of different wind directions acting on the load under luffing and slewing operation: the influence of the time-varying characteristics of wind pressure on the amplitude of the load swing angle is above 10%, and the maximum error is 47.52%.The results show that the dynamic change of wind pressure will significantly change the amplitude of the load swing, and the one-way coupling method ignores the real-time interaction between wind pressure and structural movement, resulting in a systematic deviation between the predicted value of the swing angle and the real working operations. It can be seen that the dynamic characteristics of wind pressure are the key factors to be considered in the modeling of tower crane load swing, and ignoring its role will directly affect the accuracy of the monitoring system and the effectiveness of swing suppression control.

**Table 4:** Luffing operation.

Wind direction	Radial angle amplitude			Tangential angle amplitude		
	One-way/deg	Two-way/deg	Percentage/%	One-way/deg	Two-way/deg	Percentage/%
180°	3.99	5.02	25.80	0.05	0.40	–
90°	2.72	2.11	22.43	2.12	2.37	11.79
45°	1.41	0.74	47.52	2.56	2.13	16.80

**Table 5:** Slewing operation.

Wind direction	Radial angle amplitude			Tangential angle amplitude		
	One-way/deg	Two-way/deg	Percentage/%	One-way/deg	Two-way/deg	Percentage/%
180°	1.60	2.18	36.25	3.26	2.75	15.64
90°	0.09	0.27	–	3.12	2.24	28.21
45°	2.30	1.51	34.35	3.21	2.43	24.30

#### 3.2.2. Effects of tower jib wind-induced vibration on load swing.

**Fig 17:**
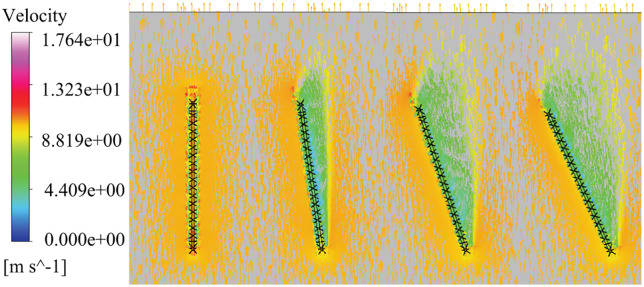
Vector Plot of the wind excitation of the tower jib.

During the slewing operation of the tower jib under stochastic wind excitation, the surrounding flow field is reconstructed (show in Fig 17), Under the transverse wind excitation with a wind speed of 10 m/s, the windward surface in the initial stage of the tower jib is directly subject to wind pressure, and the air flow is squeezed by the shape of the structure, resulting in an increase in the local flow rate. This phenomenon is particularly significant in the end area of the tower jib, forming a high-speed flow zone, which in turn causes structural deformation at the end of the tower jib. As the slewing angle increases, the windward area gradually increases, causing the airflow path to change, and the leeward area forms a flow separation zone with a significant velocity gradient, and induces periodic eddy current shedding behind the end. The eddy current movement causes the wind vibration response at the end of the tower jib, and the vibration energy is transmitted to the load end through the wire rope system, which has a secondary excitation effect on the load swing, which ultimately leads to an unexpected offset in the load swing trajectory. During the whole process, the position, intensity and flow field structure of the wind excitation showed significant nonlinear characteristics with the change of slewing angle.

**Fig 18:**
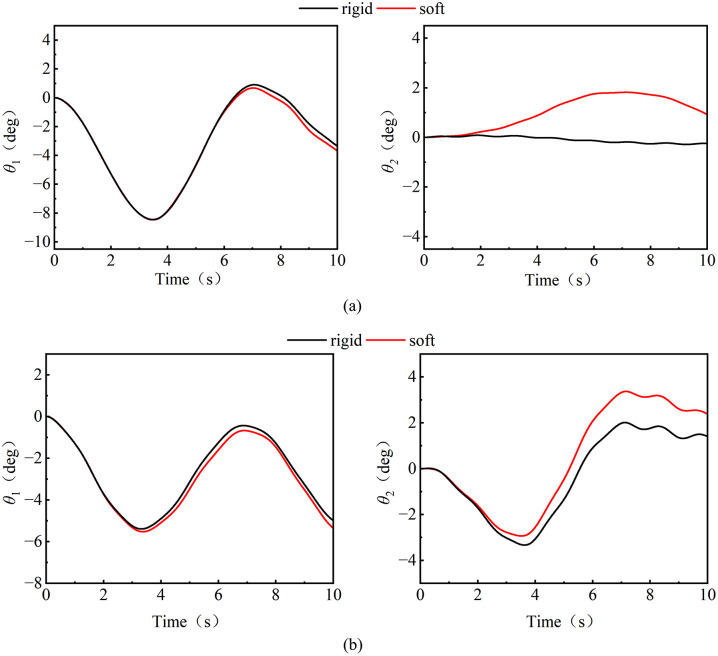
The load swing angle under the rigid and soft jib. **(a)** Luffing operation; **(b)** Slewing operation.

In order to explore the secondary excitation effect of the wind vibration response of the soft tower jib on the load swing, the tower jib was set to be a soft body and a rigid body for comparative analysis. Fig 18 shows the comparison of the load swing trajectory of the soft tower jib and the rigid tower jib during luffing and slewing operations. The results show that the vibration of the soft tower jib will have a secondary excitation effect on the amplitude of the load swing angle: The tangential swing of the load under the two operating conditions is significantly aggravated. Under the luffing operation(Fig 18(a)), the soft tower jib is affected by both wind load and axial pressure of the trolley, deformation of the tower jib by force causes the load tangential swing angle to surge by 1.53°. Under the slewing operation(Fig 18(b)), the soft tower jib can withstand both wind load and transverse bending moment, so that the maximum swing of the load is offset by 1.42°, which is 17.6% offset compared to the rigid tower jib condition (1.19°); in contrast, the radial swing angle change is small, and the mean swing angle change is 0. About 15°.Studies have shown that the elastic potential energy accumulated by the flexible deformation of the tower jib under wind excitation is released in a directional manner during the deceleration stage, forming a secondary excitation effect through the tangential dimension, resulting in a significant shift in the swing angle. The dynamic coupling mechanism makes the rigid model have a large error in the analysis of the load swing angle of the tower crane under wind excitation, and confirms the necessity of the flexible characteristics of the tower jib in the multi-body dynamic modeling of the tower crane.

### 3.3. Swing behavior of load subject to stochastic wind excitation

In this section, the nonlinear behavior of the load swing is investigated with respect to three key factors: operational modes, wind speed, and wind direction.

#### 3.3.1. Different working operations.

The operational mode of a tower crane fundamentally dictates its dynamic interaction with stochastic wind loads. This section investigates its influence from two interdependent perspectives: (1) the type of motion (e.g., luffing-only, slewing-only, and compound operations), which determines the basic kinematic excitation and coupling mechanisms; (2) the key operational parameters, specifically the luffing and slewing speeds. Variations in these speeds not only alter the intensity of excitation but also serve as effective proxies for simulating the performance characteristics of different crane types under analogous working conditions, thereby addressing the need to generalize findings beyond a single specific model.

**Fig 19:**
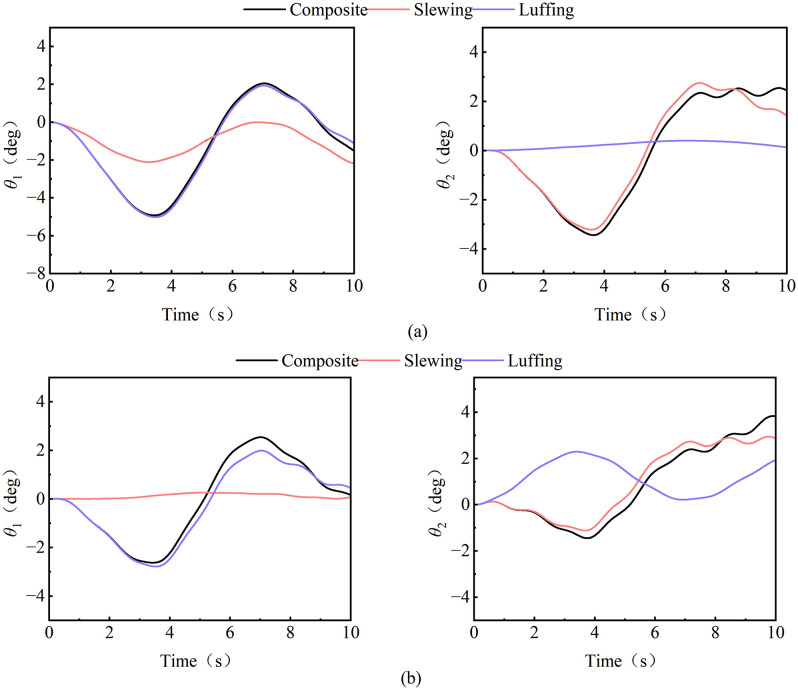
Load swing behavior of composite operation and single working operations. **(a)** Horizontal wind excitation; **(b)** Lateral wind excitation.

Through the comparative analysis of the composite operation of the tower crane under horizontal and lateral wind excitation and the load swing angle of a single operation (luffing, slewing) under horizontal and lateral wind excitation (Fig 19(a)), it is found that under horizontal wind excitation, the radial swing angle curves of the composite working conditions and luffing show quasi-sinusoidal fluctuation characteristics, with amplitudes of 3.45° and 3.48°, respectively, and the steady-state mean of the swing angle are −1.18° and −1.22°, respectively, which shows that the radial swing angle trajectories of the load under these two working operations are highly consistent; At the same time, under these two working operations, the radial swing angle trajectories of the load show a high degree of consistency; at the same time, under these two working operations The amplitude of the radial swing angle of the load should be significantly greater than the swing of 1.09° in the slewing operation; in the tangential dynamic response of the load, the swing frequency of the composite operation and the slewing operation are synchronized, the amplitude is 2.99° and 2.98°, respectively, the average swing angle is −0.06° and 0.03°, respectively, and the luffing operation moves laterally and is affected by the transverse wind excitation, so the tangential swing angle of the luffing operation is only affected by the nonlinear coupling dynamics effect, the amplitude is only 0.2°, it can be seen that the tangential swing angle of the load under these two operation is 0.06° and 0.03°, respectively. The trajectory shows significant homology. The above analysis shows that under the action of transverse wind excitation, the radial response of the composite operation is dominated by the luffing motion, while the tangential response is mainly derived from the slewing motion component.

Further analyze the load swing behavior under lateral wind excitation (as shown in Fig 19(b)), and it is found that the radial swing angle of the composite operation is similar to the luffing operation, the tangential swing angle is similar to slewing operation, which is consistent with the above obtained regularity under the action of lateral wind load. However, it can be seen that in the load tangential response under the action of lateral wind excitation, the luffing operation is completely opposite to the trend of the load swing angle under the other two operation. The main reason for this is because the load tends to move tangentially during composite and slewing operation, and there is a coupling effect between inertial force and wind excitation. However, during luffing operations, the load only has a radial movement trend, and the tangential upward is directly driven by the wind excitation, resulting in the opposite movement trend under the lateral wind excitation.

**Fig 20:**
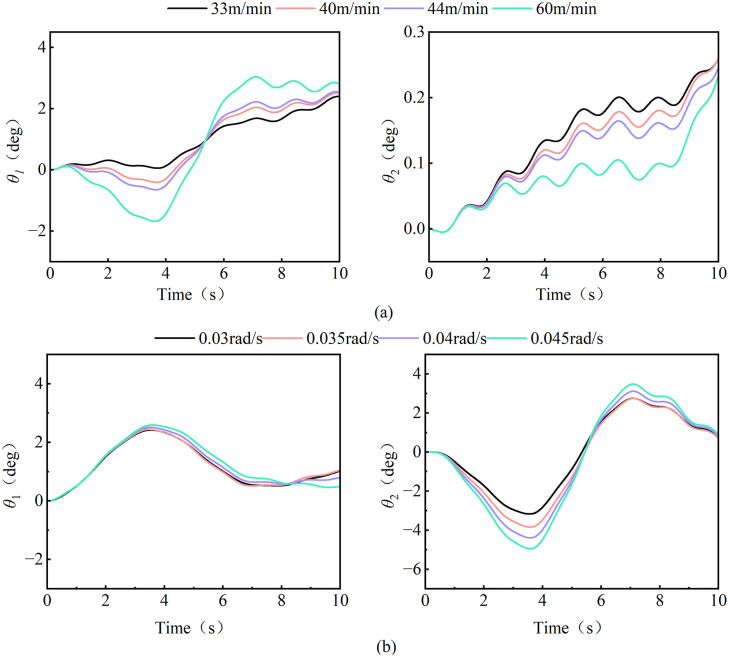
Load swing behavior under different luffing and slewing speeds. **(a)** Luffing operation; **(b)** Slewing operation.

The data from the Fig 20 reveal that the load swing of the tower crane exhibits distinct operational-mode-dependent behavior and pronounced speed sensitivity. During luffing operations (as shown in Fig 20 (a)), the radial swing angle constitutes the dominant component. Its amplitude significantly exceeds that of the tangential swing angle and increases markedly with higher luffing speeds. This indicates that luffing motion is the primary excitation source for radial load swing. Conversely, during slewing operations (as shown in Fig 20(b)), the tangential swing angle becomes the predominant response, reflecting the effects of Coriolis and centrifugal forces induced by rotational motion. More critically, high-speed conditions not only linearly amplify the steady-state swing amplitude but also trigger more complex nonlinear transient responses, such as shortened swing periods and increased overshoot. This underscores that the system’s dynamic characteristics are intrinsically linked to and vary with operational parameters, presenting a significant challenge to fixed-parameter controllers.

Therefore, the dynamic characteristics of composite tower crane operations can be effectively approximated by superimposing the load swing responses from individual luffing or slewing motions. This principle allows the control system to predict the behavior of complex combined operations using parameters identified from simpler, single-degree-of-freedom scenarios, thereby simplifying the control parameterization process while maintaining predictive accuracy. Building on this, the operational speed—a key parameter that also reflects the performance signature of different crane types—becomes critical. The system must, therefore, incorporate crane-type recognition and parameter self-tuning. By matching the crane (via its rated speed) and loading a corresponding pre-set control strategy—such as applying higher damping for high-speed models and more conservative gains for low-speed ones—the controller actively adapts to the specific dynamics. This dual approach, combining operational decoupling with speed-based adaptation, transitions control design from a single fixed solution to a versatile platform, enhancing both its applicability across a model series and its deployment efficiency.

#### 3.3.2. Different wind speeds.

**Fig 21:**
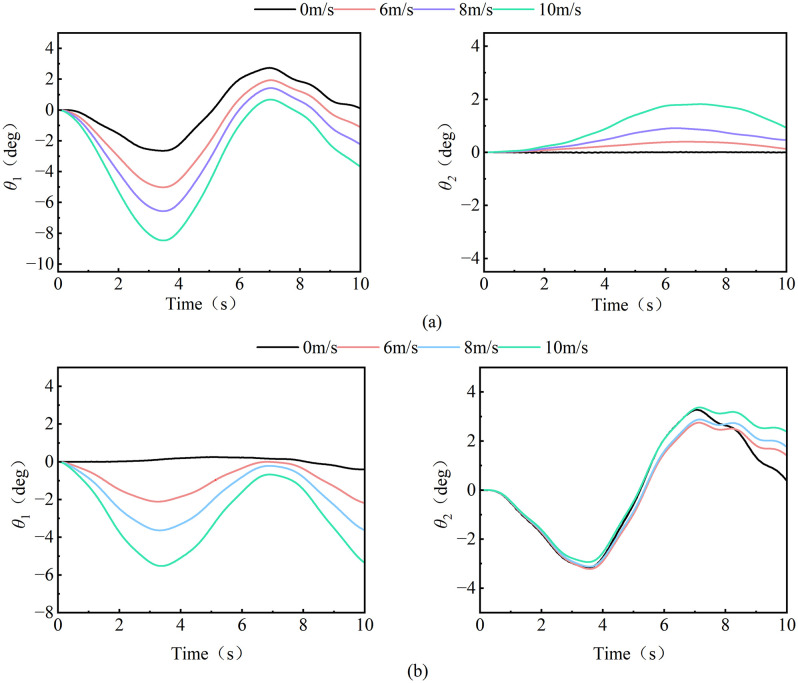
Load swing behavior at different wind speed. **(a)** Luffing operation; **(b)** Slewing operation.

**Fig 22:**
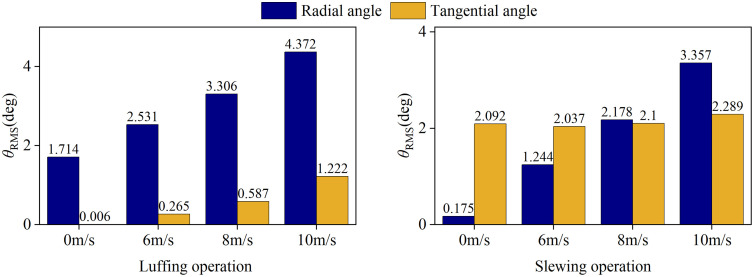
RMS diagram of load swing angle at different wind speed.

Based on the wind speed limit of China’s hoisting safety regulations for aerial operations [21] (Level 6 wind conditions: 10.8-13.8m/s, set four sets of control working conditions under the horizontal wind excitation (wind direction angle 180°): reference working conditions (0 m/s), critical early warning working conditions (6 m/s), transition working conditions (8 m/s) and working conditions before the mandatory shutdown threshold (10 m/s).Through the parameterized analysis of load dynamic response and spatial trajectory plane projection under different operating conditions (Figs 21-22), the nonlinear evolution behavior of load swing angle and displacement trajectory under characteristic wind speed (6 m/s, 10 m/s, 13 m/s) is revealed.

The Fig 21 shows that when the transverse wind excitation gradually increases from no wind to wind speed to 10 m/s, the radial swing of the load in the luffing and slewing operation increases significantly. Among them, the radial swing amplitude of the load in the luffing operation gradually increases from 2.69° to 7.81°, an increase of 1.92 times, while the slewing operation are almost stable. The 0.19° suddenly increased to 5.53°; Further comparison of the characteristics of the tangential swing angle found that the non-linear coupling effect in the luffing operation continued to accumulate with the increase of wind speed, resulting in a tangential swing angle amplitude from 0.69° to 7.81°, an increase of 1.92 times. 43° climbed to 1.81°, and the load tangential swing angle of the slewing working operation is less affected by the wind speed. In the deceleration stage, the curve is upturned, and the warping amplitude is about 0.92°. This phenomenon is directly related to the inertial force mutation of the slewing mechanism when braking.

By calculating the RMS value of the tower crane load swing under different wind excitation conditions, the damping control gain (e.g., the differential term coefficient in PID) can be dynamically adjusted. When affected by the wind excitation, the RMS value is calculated and reduced to suppress the disturbance of stochastic wind excitation to the load. From Fig 22, it can be seen that the RMS value of the radial swing angle of the luffing and slewing operation is positively correlated with the wind speed. When the wind speed reaches 10m/s, it reaches 4.372° and 3.357°, respectively. The difference between the two was 23.2%. This phenomenon shows that the sensitivity of luffing motion to wind speed disturbance is higher than that of slewing motion, and a more violent load swing response will be produced under the same wind excitation.

#### 3.3.3. Different wind directions.

**Fig 23:**
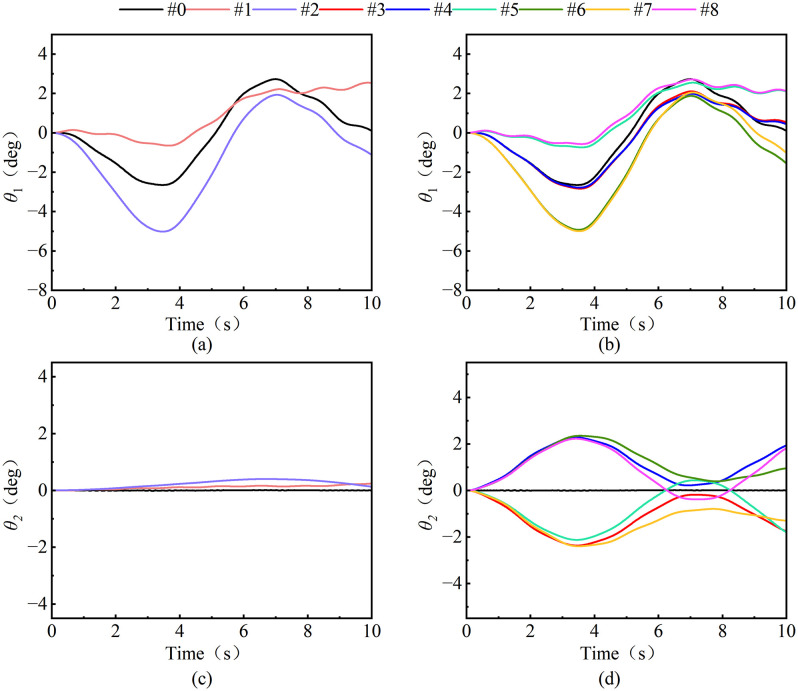
Load swing behavior under luffing operation.

**Fig 24:**
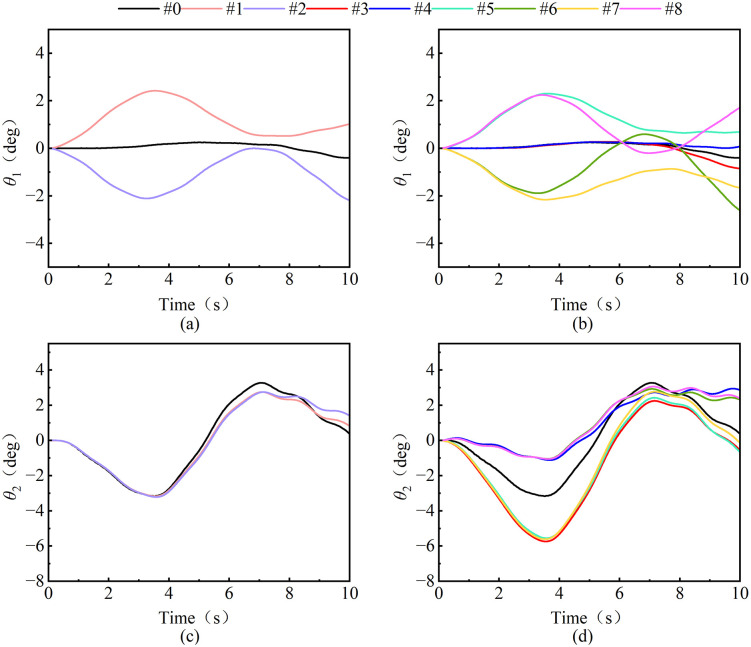
Load swing behavior under slewing operation.

**Fig 25:**
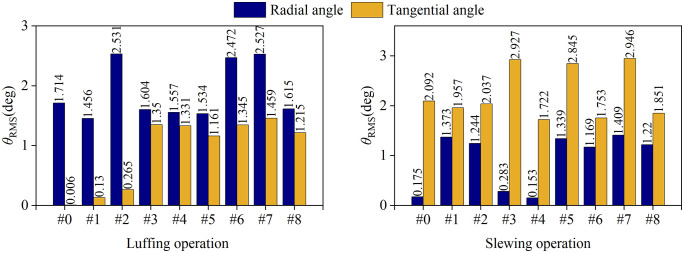
RMS diagram of load swing angle at different wind direction.

Based on the CFD/CSD two-way fluid-solid coupling analysis framework, 8 sets of typical wind excitation conditions (Table 6) are set, the wind speed is controlled to be constant (10 m/s), and the influence of the wind excitation vector direction on the evolution characteristics of the load swing trajectory is explored. Figs 23–25 reveals the nonlinear response characteristics of the load during luffing and slewing operation through the three-dimensional motion decoupling algorithm.

**Table 6:** Corresponding names of wind direction angles. (#0 means no wind condition).

Name	wind direction angles	Name	wind direction angles
#1	0°	#2	180°
#3	90°	#4	−90°
#5	45°	#6	−135
#7	135°	#8	−90°

Analysis of Fig 23 shows that during the luffing operation, the wind direction of the tower crane significantly affects the load swing angle. Fig 23(a) and Fig 23(b) show the radial swing angle of the load changes in the opposite direction when driving downwind and against the wind, while the lateral wind load on the diagonal line (e.g., 45° and −135°) has highly similar swing angle curve characteristics. The radial swing angle of the load is presented: when luffing downwind (e.g., 0°, ± 45°), the acceleration stage suppresses the swing, while the deceleration stage intensifies the swing, and when luffing upwind (e.g., 180°, ± 135°), the acceleration stage intensifies the swing, while the deceleration stage suppresses the swing; in addition, the tangential swing angle of the load(as shown in Fig 23(c) and Fig 23(d)) under the wind load direction has a slight single-sided deviation, and the swing suppression phenomenon of the radial swing angle of the wind load in the direction of ±90° starts to decrease after 5 s. In terms of load tangential swing angle: under lateral wind load (e.g., ± 45°) symmetrical along the tower jib, the load tangential swing angle trajectory is highly symmetrical along the horizontal line, with a symmetry of up to 99.8, and the tangential swing frequency of load under wind load (e.g., 45°, 90°, 135°) in the same direction.

In Fig 24 the load swing angle under the slewing operation shows a change behavior similar to the luffing operation under different wind directions. However, the swing angle behavior still differs. The radial swing angle of the load shows: Under wind directions that are symmetrical diagonally along 90° (e.g., 0° and 180°, 45° and 180°), the tangential swing trajectory of the load is highly symmetrical along the horizontal line. The tangential swing angle of the load is: when slewing downwind (−45°, −90°, and −135°), the acceleration stage suppresses the swing, while the deceleration stage intensifies the swing; when slewing upwind (45°, 90°, and 135°), the acceleration stage intensifies the swing, while the deceleration stage suppresses the swing.

The RMS distribution behavior of the load swing angle under the luffing and slewing operation of different wind directions is shown in Fig 25. It can be seen from the luffing operation: the load radial angle *θ*_RMS_ of the headwind direction (#2, #6, #7) (an average of about 2.52°) is significantly higher than that of other wind directions (an average of about 1.65°), while the radial angle *θ*_RMS_ of the load (an average of about 1.55°) in the downwind direction and the longitudinal wind excitation direction is slightly lower than in the windless environment (1.72°).This phenomenon stems from the vector superposition of the inertial force of the load and the pressure of the wind excitation during the headwind luffing operation, which significantly amplifies the dynamic response of the load swing. At the same time, the load swing angle *θ*_RMS_ under the action of lateral wind excitation (including longitudinal and oblique wind excitation) are not much different, both of which are about 1.2°.

From the slewing operation shows a similar regularity to the luffing operation, which is in the direction of the headwind. (#3, #5, #7) The tangential swing angle of the load (average of about 2.92) is 57.8% higher than that of other wind directions (average of about 1.85), while the radial swing angle of the load (average of about 1.85°) in the downwind direction and the longitudinal wind excitation direction is slightly reduced compared to the windless environment (2.09°). The reason is consistent with the luffing operation. However, under the action of its longitudinal wind excitation (#3, #4), the radial swing angle has hardly changed compared to no wind, while other wind directions have increased significantly.

Therefore, when the tower crane luffing against the wind, the radial swing of the load increases and the swing downwind decreases; when the tower crane slewing against the wind, the tangential swing of the load increases and the swing downwind decreases. Therefore, according to the recommendations shown in the Table 7, a real-time wind field perception module is introduced in the tower smart energy control system. During tower crane operation, the environmental wind excitation and wind direction can be judged in real time, and the control parameters can be dynamically adjusted to effectively suppress the windward swing of the load.

**Table 7:** Wind direction of hazardous operations.

Wind direction	Hazardous working conditions	$\theta_{RMS}$	angle direction
135° ~ −135°	Headwind luffing operation	2.52°	radial swing angle
45° ~ 135°	Headwind slewing operation	2.92°	tangential swing angle

#### 3.3.4. Load radial/tangential swing angle coupling characteristics.

In view of the nonlinear characteristics of the radial and tangential swing angles of the load under the multi-directional wind excitation of the tower crane, the Kendal rank correlation coefficient (*Kendall*) is used to quantify the coupling relationship between the radial/tangential dual-degree-of-freedom swing angle [29].The expression of the correlation coefficient is:


$$\begin{array}{c} P=\frac{\text{2}\cdot M\cdot (C-D)}{N^{\text{2}}\cdot (M-\text{1})}\; \end{array}$$ (17)

Where: *C* is the logarithm of the element with consistency in XY (the two elements are a pair); *D* is the logarithm of the element with inconsistency in XY; *N* is the number of elements; *M* is the smaller of the number of columns and rows in XY. The correlation coefficient has a range of [−1,1], where |*P*| > 0.5 characterizes a strong correlation (positive/negative) and |*P*| < 0.5 is a weak correlation.

**Fig 26:**
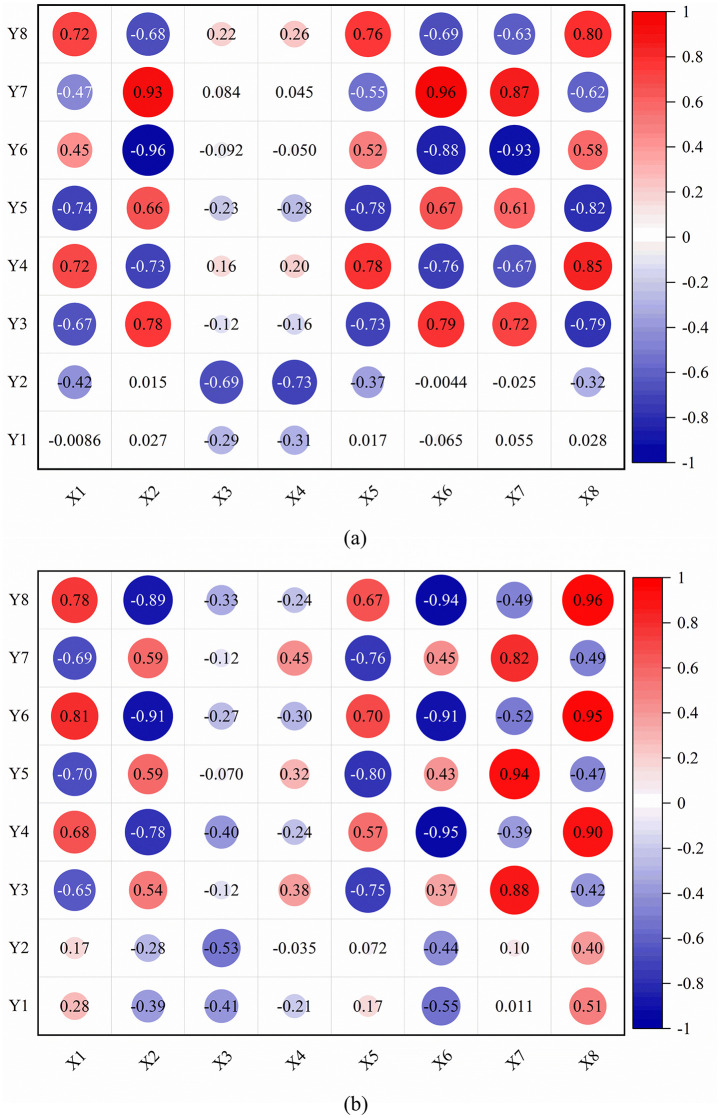
Load radial/tangential swing angle correlation. **(a)** Luffing operation; **(b)** Slewing operation. (where X and Y represent the radial swing angle and the tangential swing angle, and the number 1,2,3… indicate the wind direction at different angles, refer to Table 6.).

Through the correlation analysis of the radial/tangential swing angle changes of the load swing angle during luffing/slewing operation of the tower crane under different wind directions, the system reveals the coupling dynamics characteristics of the swing angle changes of the radial/tangential load during luffing/slewing operation of the tower crane under the full wind direction. The analysis results are shown in Fig 26, where X and Y represent the radial swing angle and the tangential swing angle, and the number 1,2,3… indicate the wind direction at different angles, refer to Table 6. For those with strong positive correlation (*P* ≥ 0.6), such as the strong positive correlation between 135° and −45° wind direction (*P* ≥ 0.8), the behavior of synergistic change of swing angle can help construct a forward gain control strategy to achieve synchronous optimization of anti-swing control parameters in different wind directions; for those with strong negative correlation (*P* ≤ −0.6), such as 45° and −135° wind direction showed a significant negative correlation (*P* ≤ −0.75), this reverse change requires complementary adjustment of the swing angle of the two degrees of freedom during monitoring and regulation to achieve different swing. Dynamic suppression between angle changes.

From the correlation analysis of the radial/tangential swing angle change of the total wind direction load, it can be concluded that the adaptive control law of “positive correlation collaborative regulation and negative correlation complementary regulation” provides a solution with both theoretical depth and engineering operability for the precise regulation of the nonlinear dynamic response of the tower crane load under stochastic wind excitation.

## 4. Conclusions

This study takes QTZ55 tower crane as the research object, based on the CFD/CSD two-way fluid-solid coupling analysis framework, comprehensively considers the flexible vibration of the jib and the dynamic evolution characteristics of wind pressure, studies the nonlinear time-varying characteristics of wind pressure and the mechanism of the influence of wind vibration of flexible structures on load swing, reveals the behavior of wind speed and wind direction changes the load swing angle, and at the same time performs a coupling analysis of the trend of load radial/tangential swing angle under different wind directions. The specific conclusions are as follows:

(1) The time-varying characteristics of wind pressure have a significant impact on the load swing: the contribution rate of the time-varying characteristics of wind pressure on the windward surface to the amplitude of the load swing angle exceeds 10% (the maximum error is 47.52%).
(2) The wind vibration response of the flexible tower jib wind is responding to the secondary excitation effect on the load swing, causing a quadratic offset of the tangential swing angle (the maximum offset is 1.82°).
(3) The change in the load swing angle is positively correlated with the wind speed; when the wind speed reaches 10m/s, the maximum load swing angle offset in the amplitude and slewing operation reaches 5.12° and 5.81° respectively.
(4) Symmetrical wind excitation (e.g., ± 45°) induces near-perfect spatial symmetry between tangential swing angle during luffing operation and radial swing angle during slewing operation, with symmetry fidelity exceeding 99.8% in dynamic simulations; and the load radial swing angle during luffing and the load tangential swing angle during slewing under different wind directions have the following behavior: When operating downwind, the acceleration stage suppresses the swing, and the deceleration stage intensifies the swing, but the load *θ*_RMS_ decreases; when operating upwind, the acceleration stage intensifies the swing, and the deceleration stage suppresses the swing, but the load *θ*_RMS_ increases.
(5) The radial and tangential swing angle changes of the load under oblique wind excitation show strong coupling characteristics. The radial/tangential swing angle of the load under the two wind directions on the 45° diagonal has a synergistic trend (*P* ≥ 0.75), while the radial and tangential swing angle of the load under the two wind directions on the −45° diagonal has the opposite trend (*P* ≤ −0.75).

In summary, the operation of a tower crane under stochastic wind excitation involves critical time-varying wind pressure and notable wind-induced vibration of the flexible system, both of which induce strong nonlinear coupling that significantly affects load swing. While the observed dynamic characteristics—such as the symmetry of swing angle under full wind directions and the coupling between tangential and radial components—offer valuable insights for anti-swing control and trajectory planning, the high degree of nonlinearity also poses a challenge for controller synthesis. To systematically address this, our next research step will employ Computational Modal Analysis (CMA) to extract the inherent linear modal parameters of the structural model. By performing a systematic comparison with the nonlinear transient results, we aim to decouple the effects of nonlinear interaction from the inherent structural dynamics. This decoupling will provide a clearer dynamic basis and facilitate the design of model-based controllers. Building on this, the CFD/CSD coupled simulation framework developed herein will serve as a high-fidelity testbed for integrating and evaluating advanced control algorithms—such as sliding mode control and adaptive control—toward the development of intelligent real-time swing suppression systems that are both dynamically informed and robust under realistic operating conditions.

To bridge the gap between academic research and practical application, validating the simulation results against scaled wind tunnel tests or field validation is crucial. Such validation is indispensable to assess the model’s robustness and predictive fidelity under real-world conditions, which will, in turn, provide critical insights for refining control strategies. Consequently, this validation constitutes the central objective of our subsequent research, with the ultimate goal of ensuring safer and more precise operation of tower cranes in complex wind environments.

## Supporting information

S1 File Contains all raw experimental data used for the statistical analyses presented in Figs 12, 14, and Tables 4, 5. (OPJU)

S2 File Contains all raw experimental data used for the statistical analyses presented in Fig 19. (OPJU)

S3 File Contains all raw experimental data used for the statistical analyses presented in Figs 18(a), 20(a), 21(a), 22–23, 25, 26(a). (OPJU)

S4 File Contains all raw experimental data used for the statistical analyses presented in Figs 18(b), 20(b), 21(b), 23–25, 26(b). (OPJU)
